# TMBIM5 loss of function alters mitochondrial matrix ion homeostasis and causes a skeletal myopathy

**DOI:** 10.26508/lsa.202201478

**Published:** 2022-06-17

**Authors:** Li Zhang, Felicia Dietsche, Bruno Seitaj, Liliana Rojas-Charry, Nadina Latchman, Dhanendra Tomar, Rob CI Wüst, Alexander Nickel, Katrin BM Frauenknecht, Benedikt Schoser, Sven Schumann, Michael J Schmeisser, Johannes vom Berg, Thorsten Buch, Stefanie Finger, Philip Wenzel, Christoph Maack, John W Elrod, Jan B Parys, Geert Bultynck, Axel Methner

**Affiliations:** 1 Institute for Molecular Medicine, University Medical Center of the Johannes Gutenberg-University Mainz, Mainz, Germany; 2 Department of Cellular and Molecular Medicine, KU Leuven, Laboratory of Molecular and Cellular Signaling, Leuven, Belgium; 3 Center for Translational Medicine, Lewis Katz School of Medicine at Temple University, Philadelphia, PA, USA; 4 Laboratory for Myology, Department of Human Movement Sciences, Faculty of Behavioural and Movement Sciences, Vrije Universiteit Amsterdam, Amsterdam, The Netherlands; 5 Department of Translational Research, Comprehensive Heart Failure Center (CHFC), University Clinic Würzburg, Würzburg, Germany; 6 Institute of Neuropathology, University Medical Center of the Johannes Gutenberg-University Mainz, Mainz, Germany; 7 Friedrich-Baur-Institute, Department of Neurology, LMU Clinic, Munich, Germany; 8 Institute of Anatomy, University Medical Center of the Johannes Gutenberg-University Mainz, Mainz, Germany; 9 Institute of Laboratory Animal Science, University of Zurich, Zürich, Switzerland; 10 Center for Thrombosis and Hemostasis, University Medical Center of the Johannes Gutenberg-University Mainz, Mainz, Germany; 11 Department of Cardiology, Cardiology I, University Medical Center of the Johannes Gutenberg-University Mainz, Mainz, Germany; 12 German Center for Cardiovascular Research (DZHK), Partner Site Rhine-Main, Mainz, Germany

## Abstract

Ion fluxes across the inner mitochondrial membrane control mitochondrial volume, energy production, and apoptosis. TMBIM5, a highly conserved protein with homology to putative pH-dependent ion channels, is involved in the maintenance of mitochondrial cristae architecture, ATP production, and apoptosis. Here, we demonstrate that overexpressed TMBIM5 can mediate mitochondrial calcium uptake. Under steady-state conditions, loss of TMBIM5 results in increased potassium and reduced proton levels in the mitochondrial matrix caused by attenuated exchange of these ions. To identify the in vivo consequences of TMBIM5 dysfunction, we generated mice carrying a mutation in the channel pore. These mutant mice display increased embryonic or perinatal lethality and a skeletal myopathy which strongly correlates with tissue-specific disruption of cristae architecture, early opening of the mitochondrial permeability transition pore, reduced calcium uptake capability, and mitochondrial swelling. Our results demonstrate that TMBIM5 is an essential and important part of the mitochondrial ion transport system machinery with particular importance for embryonic development and muscle function.

## Abstract (precis)

TMBIM5 deficiency reduces mitochondrial K^+^/H^+^ exchange. Mutation of the channel pore in mice destabilizes the protein and results in increased embryonic lethality and a skeletal myopathy.

## Introduction

Mitochondria generate a proton gradient (ΔpHm) and electrical gradient (Δψ_m_, the mitochondrial membrane potential) across the inner mitochondrial membrane (IMM). These gradients propel the F_0_F_1_-ATP synthase of the respiratory chain in a process called oxidative phosphorylation. The negative membrane potential also drives the passive uptake of cations through specific channels. Mitochondrial K^+^ channels include ATP-sensitive, Ca^2+^-activated, voltage-gated, and two-pore domain channels that regulate mitochondrial respiration, membrane potential, and life/death decisions (reviewed by Rotko et al [2020]). Mitochondrial Ca^2+^ uptake adapts mitochondrial ATP generation to demand by matching cellular and mitochondrial Ca^2+^ levels during cellular activity and is important for cellular Ca^2+^ signalling (reviewed by Giorgi et al [2018]). Ca^2+^ transfer across the IMM into the mitochondrial matrix occurs through the pore-forming protein mitochondrial Ca^2+^ uniporter (MCU) (Baughman et al, 2011; De Stefani et al, 2011). The MCU is part of a large protein complex, the mitochondrial Ca^2+^ uniporter complex (mtCU), which contains several MCU-interacting and MCU-regulatory proteins such as MICU1 (mitochondrial uptake 1) (Mallilankaraman et al, 2012), MICU2 (Patron et al, 2014), and the essential MCU regulator (EMRE) (Vais et al, 2016). Despite its importance, KO of *Mcu* in mice has a strain-dependent effect which ranges from a mild skeletal muscle phenotype (Pan et al, 2013) to lethality during late embryonic development (Luongo et al, 2015). These findings suggest that alternative mechanisms for Ca^2+^ entry may exist (Murphy et al, 2014; Wüst et al, 2017) such as the Ca^2+^/2H^+^ exchanger (CHE) leucine zipper and EF-hand containing transmembrane protein 1 (LETM1), which can work in both directions (Jiang et al, 2009). LETM1 apparently also mediates K^+^/H^+^ exchange (KHE) (Nowikovsky et al, 2004; Natarajan et al, 2021), and the question which ion is transported when and in which direction is still largely unsolved. As the ion with the highest intracellular concentration, excessive K^+^ flux into the mitochondrial matrix elicits osmotic effects and thus causes H_2_O influx into the mitochondrial matrix, triggering mitochondrial swelling. In line with a function in KHE, LETM1 loss of function results in mitochondrial swelling and a disrupted cristae architecture (Nowikovsky et al, 2004; Dimmer et al, 2008). Loss of LETM1 is lethal in flies (McQuibban et al, 2010) and mice (Jiang et al, 2013).

Similar to LETM1, the ubiquitously expressed protein Transmembrane Bax-Inhibitor Motif (TMBIM)-containing protein 5 (TMBIM5) is required for an intact mitochondrial cristae architecture (Oka et al, 2008), respiration, and ATP production (Seitaj et al, 2020). Both proteins are evolutionarily conserved. Ubiquitous knockdown of *dmTmbim5* in *Drosophila melanogaster* results in lethality at the pupa stage of development (Zhang et al, 2021). We hypothesized that TMBIM5 might be implicated in mitochondrial ion homeostasis because it is the mitochondrial member of a family of six evolutionarily conserved hydrophobic TMBIM proteins, with the founding member being BAX inhibitor-1 (BI-1, also known as TMBIM6) (Lisak et al, 2015). TMBIM6/BI-1 is a Ca^2+^-leak channel in the ER, whose Ca^2+^-flux properties are influenced by pH in a bell-shape manner (Chae et al, 2004; Westphalen et al, 2005; Bultynck et al, 2012, 2014; Kiviluoto et al, 2013). The crystal structure of the prokaryotic orthologue BsYetJ revealed a conserved di-aspartyl motif in its channel pore that impacts both Ca^2+^ affinity and pH dependence (Chang et al, 2014). Ca^2+^ binding of BsYetJ is inhibited by K^+^ and Na^+^ ions (Guo et al, 2019), suggesting that other ions may compete for Ca^2+^ and might be transported by BsYetJ and therefore possibly also by TMBIM5. These findings imply nonselective ion transport properties of TMBIM proteins, a feature recently described for the lysosomal TMBIM1 (also known as RECS1 [Zhao et al, 2006]) channel that can flux Ca^2+^ as well as Na^+^ (Pihán et al, 2021). Mutation of the di-aspartyl motif abolishes the Ca^2+^ flux properties of both TMBIM6 (Bultynck et al, 2012) and TMBIM1 (Pihán et al, 2021). Together these homologies suggest a role for TMBIM5 in mitochondrial Ca^2+^ transport and ion homeostasis, which we decided to investigate in vitro and in vivo.

We found that overexpressed TMBIM5 mediates mitochondrial Ca^2+^ uptake via key aspartate residues located in its channel pore. Its deficiency, however, has no drastic effect on Ca^2+^ uptake but alters the matrix ion composition with increased K^+^ and reduced H^+^ levels, coinciding with mitochondrial swelling. To address the function of TMBIM5 in vivo, we generated mice carrying a mutation in one of the aspartate residues important for its channel-pore properties. This mutation renders the TMBIM5 protein unstable. Mutant mice display increased embryonic lethality and a skeletal myopathy which strongly correlates with tissue-specific disruption of cristae architecture, early opening of the mitochondrial permeability transition pore, reduced Ca^2+^ uptake capability, and mitochondrial swelling. Together, our results imply that TMBIM5 is an essential and important part of the mitochondrial ion transport system machinery.

## Results

### Overexpressed TMBIM5 increases mitochondrial Ca^2+^ uptake

**Figure 1.**
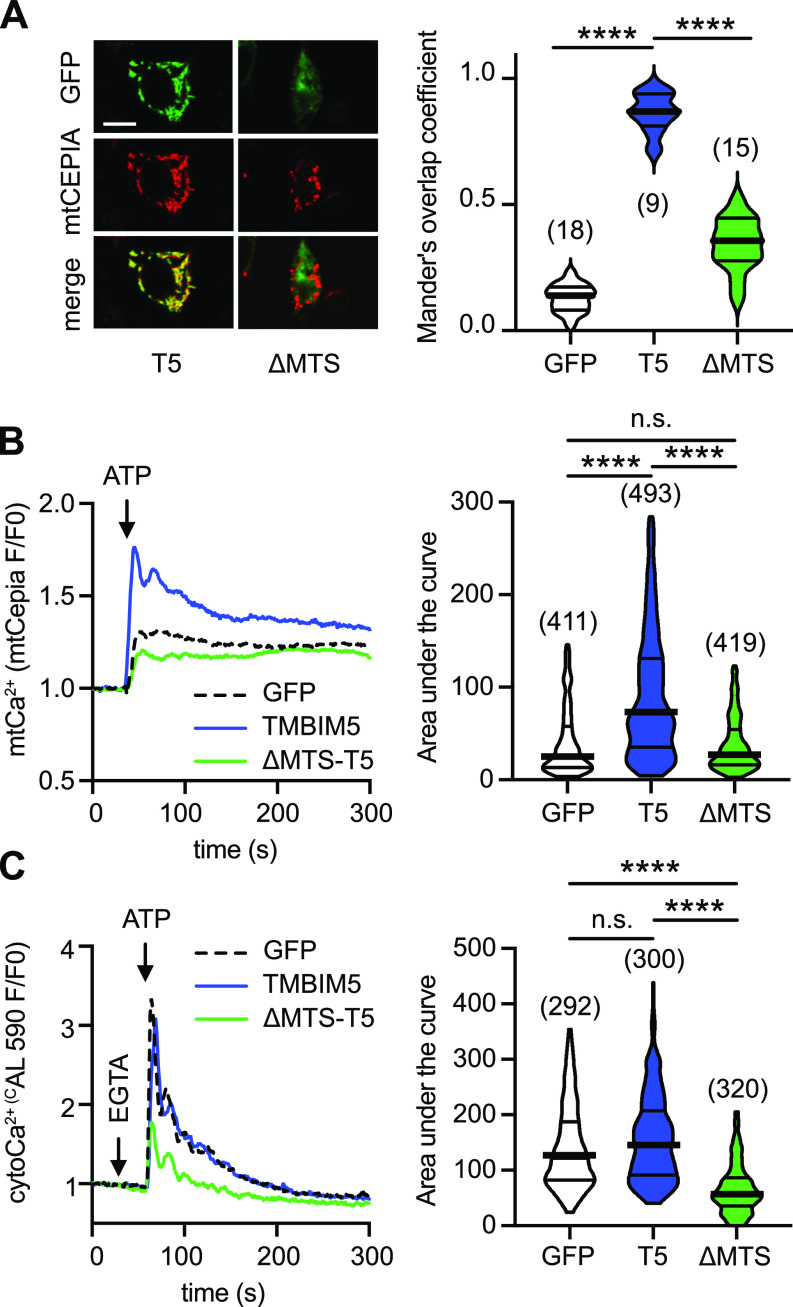
Overexpressed TMBIM5 mediates mitochondrial Ca^2+^ uptake. **(A)** Overexpression of TMBIM5-GFP (T5) but not of GFP alone or of a mutant lacking the mitochondrial targeting signal (ΔMTS) co-localizes with R-CEPIA3*mt*. Scale bar, 10 μm. Left panel shows exemplary pictures and the right panel the quantification of colocalization. **(B, C)** Overexpression of TMBIM5-GFP but not GFP or ΔMTS-T5 increases (B) mitochondrial Ca^2+^ levels measured with R-CEPIA3*mt* but not (C) cytosolic Ca^2+^ levels upon ATP-mediated Ca^2+^ release from the ER. Data are shown as violin plots with the mean and the 25th to 75th percentile indicated. Statistical significance was calculated with the Kruskal–Wallis test, n is indicated in brackets or shown as individual data points, biological replicates (N) = 3, ****P < 0.0001, n.s., nonsignificant.

Mitochondria take up Ca^2+^ released from the ER at mitochondria-ER contact sites (MERCS), hot spots of interactions between the two organelles where local Ca^2+^ concentrations reach very high levels (reviewed in Marchi et al [2014]). To determine whether TMBIM5 can mediate mitochondrial Ca^2+^ uptake, we overexpressed TMBIM5 in HEK293 cells and stimulated inositol 1,4,5-trisphosphate (IP_3_)–mediated Ca^2+^ release from the ER with ATP, an extracellular agonist of Gq-coupled metabotropic ATP receptors. We then measured the ATP-induced mitochondrial Ca^2+^ transients using the genetically encoded red Ca^2+^ sensor R-CEPIA3*mt* (Kanemaru et al, 2020) in TMBIM5-expressing cells, visualized by a C-terminal GFP tag in TMBIM5. As control, we expressed GFP alone, as well as TMBIM5 in which we removed the N-terminal mitochondrial transfer signal (ΔMTS-TMBIM5-GFP). As a consequence, ΔMTS-TMBIM5 does not traffick to the mitochondria and accumulates at the ER (Fig 1A). This fact allowed us to study an effect of TMBIM5 on the ER Ca^2+^ content, thus excluding any indirect effects on Ca^2+^ release caused by interacting proteins in the mitochondrial membrane. Measurements of mitochondrial calcium signals revealed that IP_3_ receptor stimulation provoked a significantly larger increase in mitochondrial Ca^2+^ levels in cells transfected with wild-type TMBIM5 compared with GFP alone (Fig 1B). Cytosolic Ca^2+^ increases induced by ATP, in contrast, were not affected by TMBIM5 overexpression (Fig 1C) effectively ruling out the possibility that the increased mitochondrial Ca^2+^ levels were secondary to increases in cytosolic Ca^2+^ concentration. In contrast to wild-type TMBIM5, ER-localized ΔMTS-TMBIM5 did not augment ATP-evoked mitochondrial Ca^2+^ uptake (Fig 1B), but significantly lowered IP_3_-mediated cytosolic Ca^2+^ transients (Fig 1C). This is reminiscent of the function of the ER-localized homologue BI-1/TMBIM6 (Westphalen et al, 2005; Bultynck et al, 2012), suggesting that TMBIM5 could be the functional orthologue expressed at mitochondria. Furthermore, this effect of an ectopically expressed Ca^2+^ leak channel that is directed to the ER rules out changes in the mitochondrial driving force or MERCS as an explanation for the increased mitochondrial Ca^2+^ transients in TMBIM5-transfected cells. Together, these data suggest that overexpression of TMBIM5 can mediate mitochondrial Ca^2+^ uptake under conditions of high local Ca^2+^ concentrations.

### No alteration of mitochondrial Ca^2+^ uptake in TMBIM5-deficient cells

**Figure 2.**
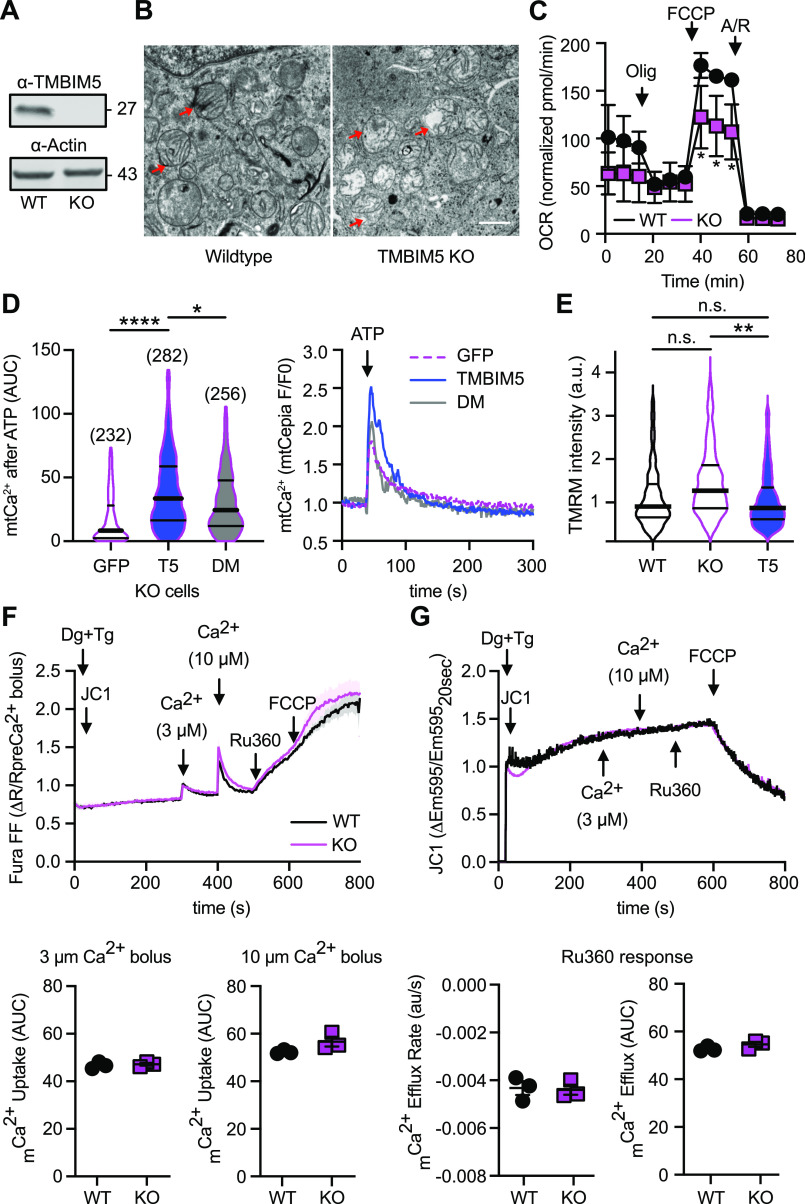
No alteration of mitochondrial Ca^2+^ uptake in TMBIM5-deficient cells. **(A)** Immunoblot demonstrating loss of TMBIM5 expression in KO HEK293 cells after CRISPR/Cas9-mediated gene inactivation. Size is indicated, β-actin served as loading control. Wild-type, WT. **(B)** Disrupted cristae architecture with ballooning cristae and cristae loss shown by transmission electron microscopy. Scale bar 1 μm. **(C)** Reduced oxygen consumption in *TMBIM5* KO cells measured in a Seahorse assay. **(D)** Overexpression of TMBIM5 (T5) but not empty vector (GFP) or TMBIM5 with point mutations in the channel pore (D294R/D325R, DM) rescues reduced mitochondrial Ca^2+^ transients measured with R-CEPIA3*mt* upon ATP-mediated Ca^2+^ release from the ER. **(E)** The reduced mitochondrial Ca^2+^ uptake in KO cells is not caused by differences in the driving force because the membrane potential measured with TMRM in a high-content microscope is similar in WT and T5-rescued KO cells. **(F, G)** Unchanged mitochondrial Ca^2+^ uptake and membrane potential in reductionist Ca^2+^ uptake assays. WT and KO cells were permeabilized with digitonin in the presence of the SERCA inhibitor thapsigargin and loaded with the radiometric Ca^2+^ sensor Fura-FF. The ratiometric mitochondrial membrane potential (∆ψ) reporter, JC-1, was added at 20 s. At 300 s, a 3 μM Ca^2+^ bolus was added followed by a 10 μM Ca^2+^ bolus at 400 s. Ru360, to inhibit the mitochondrial calcium uniporter, was added at 500 s and after 600 s, the protonophore, FCCP. Shown below from left to right: total _m_Ca^2+^ uptake (area-under the curve) post 3 μM Ca^2+^ bolus, total _m_Ca^2+^ uptake (area-under the curve) post 10 μM Ca^2+^ bolus, _m_Ca^2+^ efflux rate post Ru360 addition and total _m_Ca^2+^ efflux (area-under the curve) post Ru360 addition. **(D, E, G)** Data are shown as mean ± SE in C (n = 3), scatter plots of individual values with mean ± SE in (G), or violin plots with the mean, the 25th to 75th percentile and n indicated in (D) and (E). **(C, D, E, G)** Statistical significance was calculated with the Kruskal–Wallis (D, E) or the Mann–Whitney test (C, G). *P < 0.05; **P < 0.001; ****P < 0.0001; n.s.; nonsignificant.

To study the endogenous function of TMBIM5, we generated *TMBIM5*-deficient HEK293 cells using CRISPR/Cas9. Absence of TMBIM5 protein was confirmed by immunoblotting (Fig 2A). *TMBIM5*-deficient cells have a disrupted cristae architecture with loss of cristae (Fig 2B) and reduced maximal uncoupled mitochondrial respiration as shown by a Seahorse analysis (Fig 2C) similar to previously reported TMBIM5 loss-of-function cellular phenotypes (Oka et al, 2008; Seitaj et al, 2020; Zhang et al, 2021). Based on these findings, we concluded that our *TMBIM5* KO HEK293 cells constitute a bona fide model to study the effects of TMBIM5 loss of function on mitochondrial Ca^2+^ dynamics.

**Figure S1.**
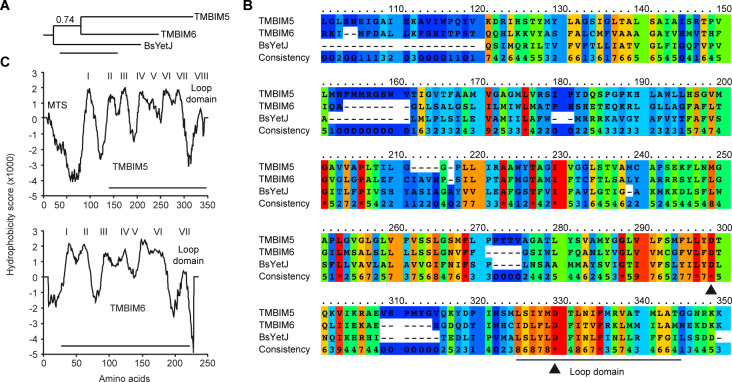
The putative loop domain is conserved between human TMBIM5, TMBIM6/BI-1, and their bacterial orthologue BsYetJ. **(A)** Phylogenetic analysis of the sequence alignment of the three proteins created using the maximum likelihood algorithm with the BLOSUM62 substitution matrix and 100 bootstrap trials. The branch support values are indicated above the branches. **(B)** TMBIM5 has a mitochondrial targeting signal (MTS) and an additional transmembrane domain I but otherwise shares the same overall structure, with the last domain being much less hydrophobic than the others. Hydrophobicity plots were generated by TMpred. Putative transmembrane domains are indicated by roman numerals. The TMBIM6 homology loop domain is indicated by a horizontal line. **(C)** Multiple protein sequence alignment of the three proteins was performed using PRALINE with the BLOSUM62 scoring matrix. The colors indicate the least conserved (blue) to the most conserved residues (red). Nonaligning N termini of each protein were removed. Arrowheads indicate key residues previously shown to be important in gating pH-dependent Ca2+ leak by BsYetJ and TMBIM6/BI-1 and the corresponding residues in TMBIM5 (D295 and D326).

To provide evidence supporting a channel activity of TMBIM5, we decided to overexpress a channel-mute mutant of TMBIM5 in these *TMBIM5* KO cells and compare its effect on ATP-evoked mitochondrial Ca^2+^ uptake with wild-type TMBIM5. We used *TMBIM5* KO cells to rule out an effect of the wild-type TMBIM5. We mutated two highly conserved negatively charged aspartate residues in the presumed pore domain (Fig S1) to neutral arginine residues. These residues were chosen because their mutation abolishes channel function in the homologous proteins TMBIM6/BI-1 (Bultynck et al, 2012) and TMBIM1/RECS1 (Pihán et al, 2021) as well as in the bacterial orthologue BsYetJ (Guo et al, 2019). Overexpression of TMBIM5 D294R/D325R (DM) was not able to increase ATP-evoked mitochondrial Ca^2+^ uptake to the same levels as overexpressing wild-type TMBIM5, implying that these aspartate residues partially disrupt TMBIM5 function, and most likely its channel activity (Fig 2D). The reduced uptake of Ca^2+^ in KO cells was not caused by a reduced driving force as the mitochondrial membrane potential measured with the dye tetramethylrhodamine (TMRM) was rather increased (Fig 2E). To rule out effects of the plasma membrane or ER Ca^2+^ exchange, we next addressed mitochondrial Ca^2+^ uptake in reductionist experiments by adding Ca^2+^ pulses to digitonin-permeabilized cells in the presence of thapsigargin, an inhibitor of sarco/ER Ca^2+^ ATPases (Fig 2F). To exclude differences in the driving force, we simultaneously measured the mitochondrial membrane potential with JC-1, which in this setup revealed no major differences between groups (Fig 2G). After the last Ca^2+^ pulse, we added Ru360, an MCU inhibitor (Woods et al, 2019) to evaluate Ca^2+^ extrusion mediated by NCLX, the electrogenic sodium-lithium exchanger, which exports Ca^2+^ in exchange for Na^+^ ions (Palty et al, 2010; Luongo et al, 2017). Finally, FCCP was added to dissipate the membrane potential and release all unbound matrix Ca^2+^. Surprisingly, these experiments revealed no significant differences in Ca^2+^ uptake and release between WT and *TMBIM5* KO cells under these conditions. There were also no differences in Ca^2+^ release after inhibition of MCU-mediated Ca^2+^ uptake with Ru360 (Fig 2G). We concluded that in this experimental setup, TMBIM5-mediated Ca^2+^ uptake is too small or too slow to be observed in the presence of an active mtCU, in line with the extensive literature describing the mtCU as the major channel for acute mitochondrial Ca^2+^ uptake.

### TMBIM5 deficiency attenuates potassium-proton exchange activity

The prokaryotic TMBIM5 orthologue BsYetJ can be inhibited by K^+^ and Na^+^ ions within the physiological range (Guo et al, 2019). In addition, another TMBIM family member, TMBIM1, can also transport Na^+^ in addition to Ca^2+^ (Pihán et al, 2021). Together, these results imply that TMBIM proteins might be nonselective ion channels that gate Ca^2+^ only under specific conditions and that other ions could influence their Ca^2+^ transport properties.

**Figure 3.**
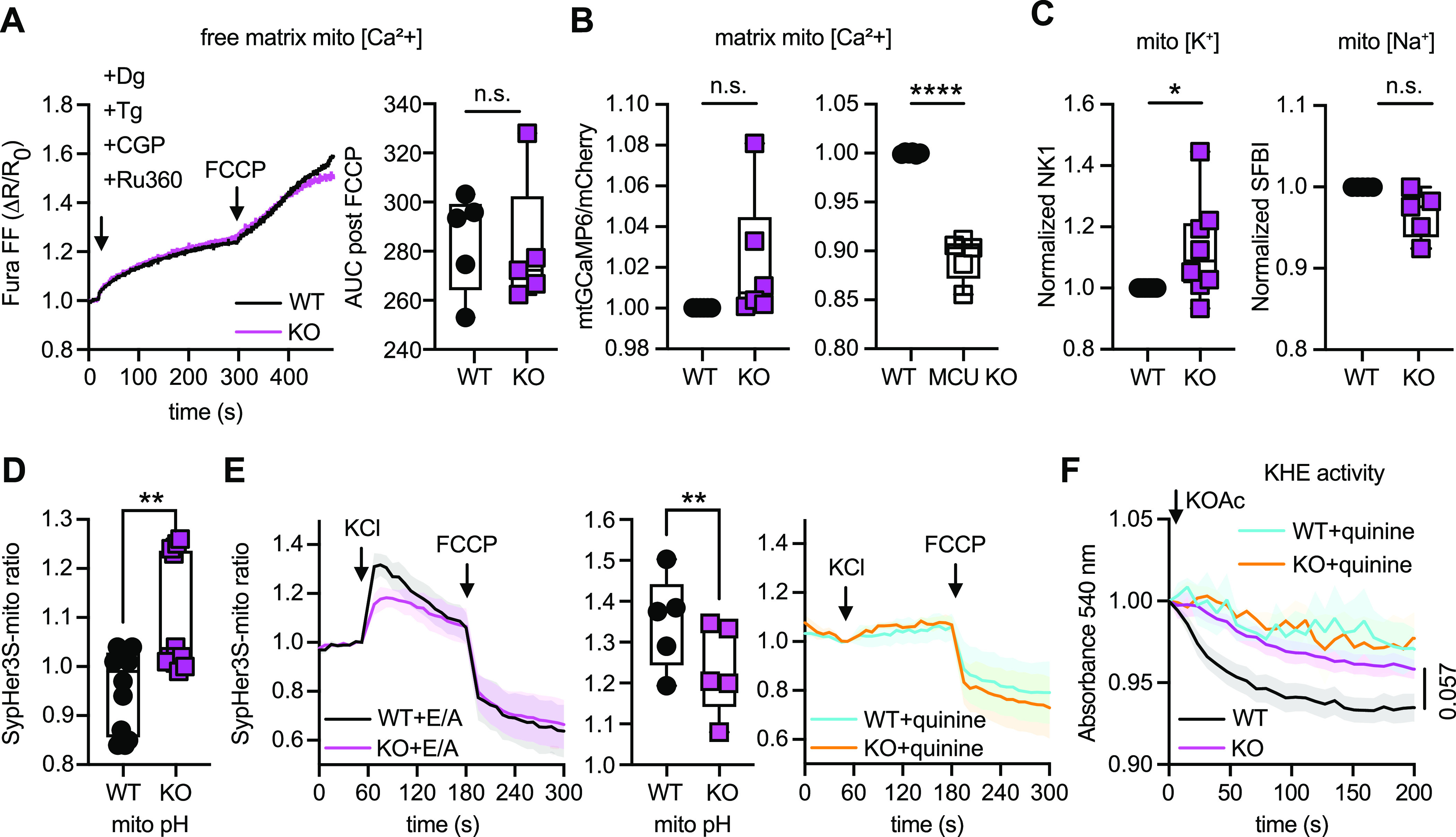
TMBIM5 deficiency alters the K^+^/H^+^ homeostasis. **(A)** Raw traces show Fura FF recordings corresponding to extramitochondrial Ca^2+^ levels to monitor matrix free Ca^2+^. Matrix free Ca^2+^ was calculated as total Ca^2+^ released from mitochondria in response to FCCP induced mitochondrial depolarization. AUC, area under the curve. **(B)** No alteration in mitochondrial [Ca^2+^] in *TMBIM5* KO cells quantitated using a ratiometric matrix Ca^2+^ sensor mito-mCherry-GCamP6. MCU KO cells served as control and have lower Ca^2+^ levels. **(C)** Increased matrix K^+^ (measured with NK1) but not Na^+^ (measured with SBFI) concentration measured using specific probes. **(D)** Reduced matrix proton concentration in *TMBIM5* KO cells determined using the pH sensor SypHer3S targeted to the mitochondrial matrix. **(E)** Reduced H^+^ extrusion in *TMBIM5* KO cells in response to 200 mM KCl added to permeabilized cells determined using the pH sensor SypHer3S targeted to the mitochondrial matrix. EDTA/A23187 (E/A) was added to enhance KHE activity. The KHE blocker quinine abolishes the response in both cell lines. E/A or quinine were added at time point 0. **(F)** Reduced KHE activity in *TMBIM5* KO cells. Mitochondrial preparations were treated with 5 μM antimycin A and 10 mM EDTA, 1 μM A23187, and the absorbance quantified immediately after adding the KOAc. In control experiments, the KHE inhibitor quinine (1 mM) was added together with EDTA/A23187. **(A, B, C, D, E)** Data were plotted as box and whisker plots with the box representing the 25–75th percentile and the whiskers indicating min to max, each dot represents an individual experimental measure (A) or the mean of one individual experiment conducted in triplicates (B, C, D, E). **(E, F)** Curves with shadow in (E) and (F) show the mean ± SE of n = 5 individual experiments. **(A, B, C, D, E)** Statistical analysis was done using the Mann–Whitney test in (A, D, E) and nested one-sample *t* tests in (B, C). *P < 0.05; **P < 0.001; ****P < 0.0001; n.s.; nonsignificant.

We therefore studied whether TMBIM5 plays a role in other mitochondrial ion transport mechanisms and queried the steady-state matrix concentrations of Ca^2+^, Na^+^, K^+^, and H^+^ ions in *TMBIM5* KO and WT cells. The steady-state matrix Ca^2+^ concentration was first measured by quantifying the amount of Ca^2+^ being released by depolarization with FCCP from mitochondria in the presence of CGP to block Ca^2+^ extrusion and Ru360 to inhibit Ca^2+^ influx. This demonstrated no difference (Fig 3A). To support this observation, we also measured the matrix Ca^2+^ concentration using a ratiometric fluorescent Ca^2+^ sensor based on mito-mCherry-GCamP6 (Stavsky et al, 2021). This yielded the same results: Ca^2+^ levels were similar in WT and *TMBIM5* KO cell lines despite a tendency towards higher levels in KO cells (Fig 3B left panel). *MCU* KO HEK293 cells served as control and these cells had significantly lower levels, demonstrating the suitability of our approach (Fig 3B right panel).

We next assessed matrix K^+^ levels in intact cells by comparing WT and *TMBIM5* KO cells stained with the NK1 sensor, which specifically localizes to mitochondria and has almost no responses to physiological pH and other biologically relevant metal ions (Ning & Tian, 2020). Matrix Na^+^ levels in isolated mitochondria were measured using benzofuran isophthalate tetra-ammonium salt (SFBI) (Hernansanz-Agustín et al, 2020). This demonstrated a significant increase in K^+^ but not Na^+^ concentration in *TMBIM5* KO cells (Fig 3C). Matrix pH in intact cells was quantified using the SypHer-3S reporter targeted to the matrix (Ermakova et al, 2018). This assay revealed a lower H^+^ concentration in *TMBIM5* KO cells compared with WT cells (Fig 3D). Because of the combination of low H^+^ and high K^+^ concentration in *TMBIM5* KO cells, we next monitored the matrix [H^+^] in response to 200 mM KCl in digitonin-permeabilized cells to assess K^+^/H^+^ exchange (KHE) activity. As this process is accelerated by the depletion of divalent cations by the chelator EDTA and the electroneutral 2H^+^-divalent metal ion (mainly Mg^2+^ and Ca^2+^) exchanger A23187 (Bernardi, 1999), both compounds were added right before the experiment. FCCP served as control because it dissipates the pH gradient. The KHE blocker quinine served as negative control. These experiments revealed a reduced KHE activity in *TMBIM5* KO cells (Fig 3E). To substantiate this, we also measured KHE activity by studying potassium acetate–based (KOAc) swelling as described (Austin et al, 2017). Mitochondria were de-energized by incubation with the complex III blocker antimycin A in the presence of KOAc medium, EDTA, and A23187. The protonated form of acetic acid accumulates in the mitochondrial matrix and catalyzes the uptake of K^+^ in exchange with H^+^ via the KHE, resulting in swelling by accumulation of matrix K^+^ acetate. The KHE inhibitor quinine served again as negative control. These experiments corroborated that *TMBIM5* KO mitochondria have a reduced KHE activity (Fig 3F). We conclude that TMBIM5 deficiency alters the matrix K^+^/H^+^ homeostasis and attenuates KHE activity.

### TMBIM5 does not associate with LETM1 in a macromolecular complex

**Figure 4.**
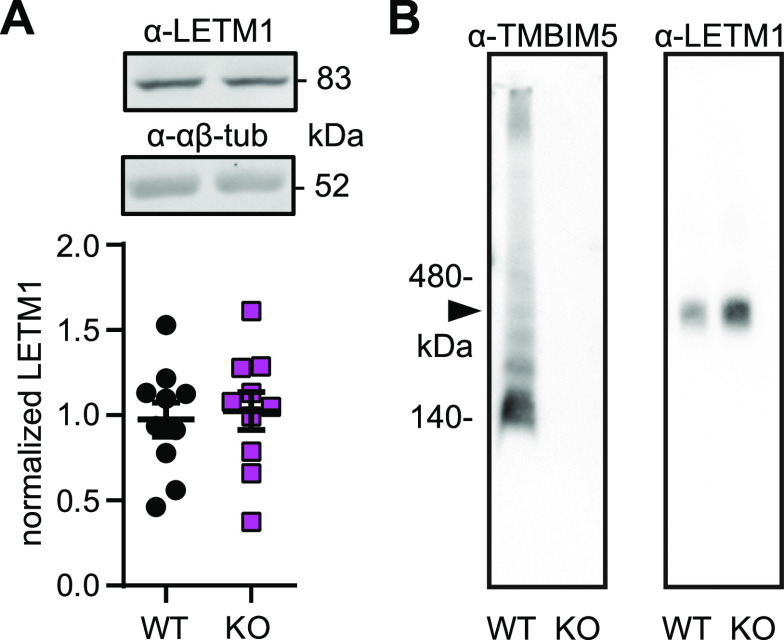
TMBIM5 does not associate with LETM1 in a macromolecular complex. **(A)** LETM1 abundance is unchanged in KO cells shown by immunoblotting. Molecular weight is indicated, actin and αβ-tubulin served as controls. **(B)** Blue native gel electrophoresis of TMBIM5 and LETM1. Molecular weight is indicated. Arrowhead indicates molecular weight of the LETM1 complex. Each dot in (A) represents one immunoblot, mean ± SEM, non significant, *t* test.

The newly identified role of TMBIM5 in K^+^/H^+^ homeostasis and in Ca^2+^ influx led us to study a potential interaction of TMBIM5 with LETM1, a proposed molecular KHE effector (Nowikovsky et al, 2004; Natarajan et al, 2021) which also affects Ca^2+^ transport (Jiang et al, 2009; Shao et al, 2016). LETM1 protein levels quantified by immunoblotting were similar between control and TMBIM5 KO cell lines, ruling out that the observed effects on KHE were due to direct changes in LETM1 abundance (Fig 4A). Blue native gel electrophoresis revealed that TMBIM5 runs mainly in a complex of about 140 kD, whereas LETM1 runs as a distinct and much larger complex that was not affected by loss of TMBIM5 (Fig 4B). We conclude that the effects of TMBIM5 on KHE are probably not mediated by changes in LETM1 abundance or complex formation.

### Mutation of the di-aspartyl motif of TMBIM5 results in increased embryonic or perinatal mortality and a skeletal myopathy in mice

**Figure 5.**
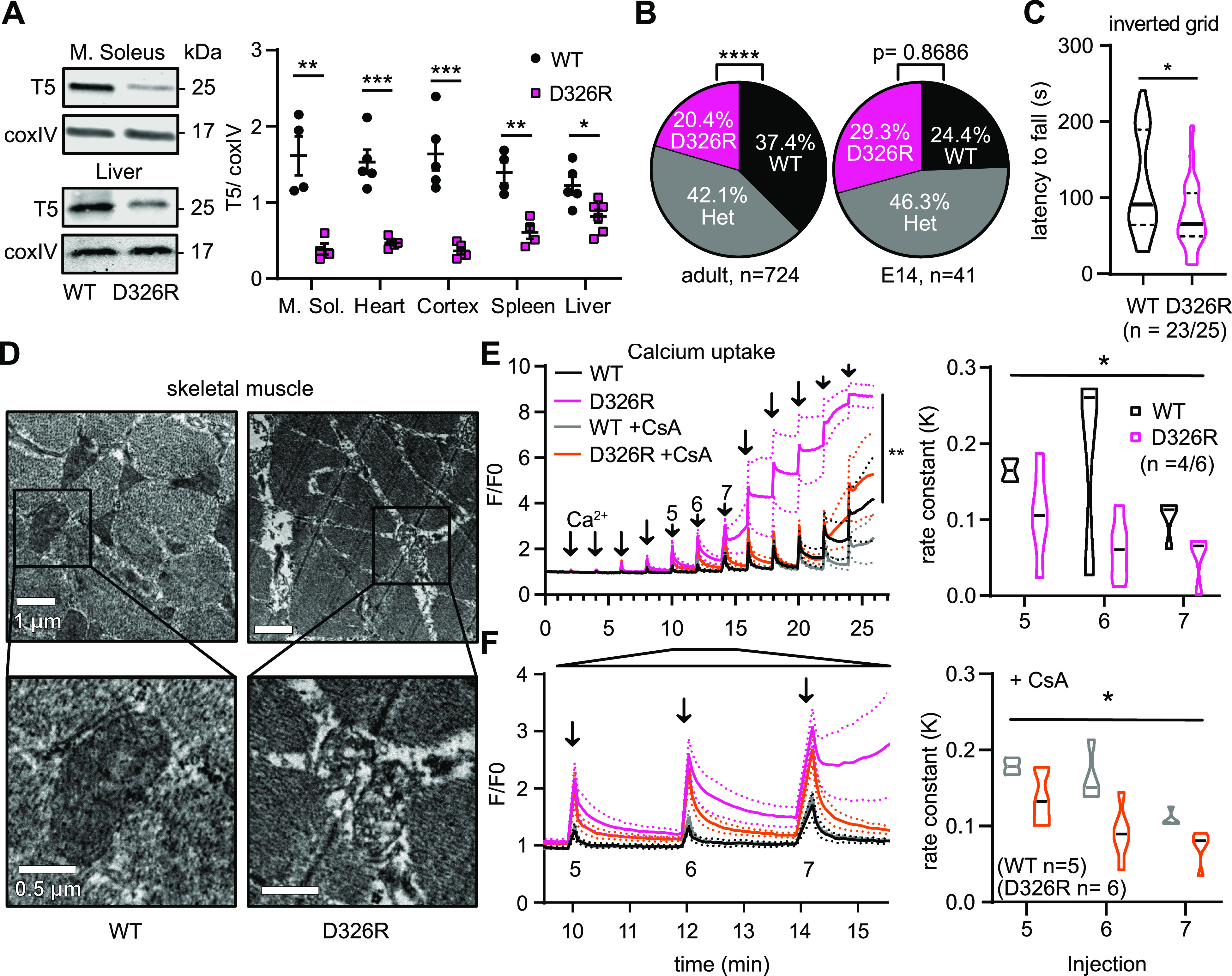
Mutation of a conserved critical residue in the presumed channel pore associates with late embryonic lethality and a skeletal myopathy in mice. **(A)** Immunoblotting of TMBIM5 demonstrates a drastic reduction of TMBIM5 protein expression in all tested tissues. CoxIV served as loading control. Size is indicated. **(B)** Reduced number of genotyped homozygous TMBIM5 D326R mice than expected from heterozygous breeding. This is not the case for E14 embryos, indicating increased lethality at a late embryonic or perinatal time-point. **(C)** Reduced muscle strength in TMBIM5 D326R mice. Mice were placed on a horizontal grid and allowed to accommodate for 2 s, then the grid was turned 180° and the latency to fall was measured (WT/D326R: n = 23/25). **(D)** Exemplary transmission electron microscopy images showing damaged internal mitochondria architecture with ballooning of cristae in D326R skeletal muscle tissue. **(E, F)** Slower Ca^2+^ uptake and reduced uptake capacity in isolated TMBIM5 D326R skeletal muscle mitochondria. Bath Ca^2+^ was measured by Calcium Green-5N. When challenged with a series of Ca^2+^ pulses (10 μM each), mPTP in D326R mitochondria opens earlier. Ca^2+^ uptake is slower in the absence or presence of 2 μM CsA. On the right, rate constant (K) of the uptake slope after the indicated Ca^2+^ injections ± CsA. Fluorescence was normalized to the initial value (F_0_). (WT ± CsA: 4/5; D326R ± CsA: n = 6). Data are shown as violin blots indicating the median ± quartiles, all other data are shown as mean ± SE. **(A, C)** unpaired *t* test, (B) Chi-square test, (E, F) mixed-effects analysis, n.s. *P* ≥ 0.05, *P < 0.05, **P < 0.01, ***P < 0.001, ****P < 0.0001.

Given the cellular phenotypes associated with TMBIM5 loss, arguing for an important role in mitochondrial ion homeostasis, we next set out to study TMBIM5 function in vivo. We decided to disrupt the channel-lining di-aspartyl motif and altered the polarity of the negatively charged aspartic acid at position 326 (corresponding to human D325) which lines the presumed channel pore of TMBIM5 and all other TMBIM proteins, including the prokaryotic BsYetJ (Chang et al, 2014; Lisak et al, 2015) (Fig S1). This amino acid is essential for the Ca^2+^ channel activity of TMBIM6 (Bultynck et al, 2012) and BsYetJ (Chang et al, 2014), as well as the Ca^2+^ and Na^+^ channel activity of TMBIM1 (Pihán et al, 2021). We replaced the aspartic acid residue with a basic arginine by CRISPR/Cas9–mediated germline mutagenesis in C57BL/6 mice, generating the C57BLB/6J-Ghitm^em1(D326R)LTK^ line, here called TMBIM5 D326R. The D326R point mutation destabilized the TMBIM5 protein and enhanced its degradation in all tissues (Fig 5A) resulting in a de facto knockdown of the channel-mute protein. Disruption of the channel function apparently renders the protein nonfunctional and prone to degradation. Altough, at embryonic day 14, the genotype distribution followed the expected Mendelian distribution, we found that homozygous TMBIM5 D326R mice were born at a lower-than-expected Mendelian ratio (Fig 5B), suggesting that TMBIM5 is involved in embryonic development or perinatal survival. The most prominent phenotype was skeletal muscle myopathy. Homozygous TMBIM5 D326R mice have reduced muscle strength, as shown by the inverted grid assay, which measures how long the mice can hold their bodyweight when turned upside down (Fig 5C). To analyze if this lack of muscle strength is due to impaired mitochondrial function, we analyzed mitochondrial ultrastructure. Thigh muscle mitochondria appeared to be swollen, exhibiting a disrupted cristae structure (Fig 5D), consistent with the cellular phenotypes of loss of TMBIM5 (Oka et al, 2008; Seitaj et al, 2020).

To test whether TMBIM5 also regulated Ca^2+^ influx in vivo, we studied the kinetics of Ca^2+^ uptake and the total Ca^2+^ retention capacity by adding repetitive pulses of Ca^2+^ to isolated skeletal mitochondria. When the maximum uptake capacity of MCU exceeds the maximum efflux velocity, high mitochondrial Ca^2+^ concentrations lead to opening of the mitochondrial permeability transition pore (mPTP), a nonselective high-conductance pore that dissipates mitochondrial membrane potential. This demonstrated a faster opening of the mPTP in D326R mitochondria (Fig 5E) suggesting an enhanced susceptibility of mutant mitochondria to this insult in line with the increased susceptibility to apoptosis and cytochrome *c* release in TMBIM5 loss-of-function cell models (Oka et al, 2008; Seitaj et al, 2020). When opening of the mPTP was inhibited by cyclosporine A (CsA) (Broekemeier et al, 1989), Ca^2+^ uptake was reduced in mutant D326R mitochondria (Fig 5F) in line with the Ca^2+^ transport capability of heterologously over-expressed TMBIM5.

**Figure 6.**
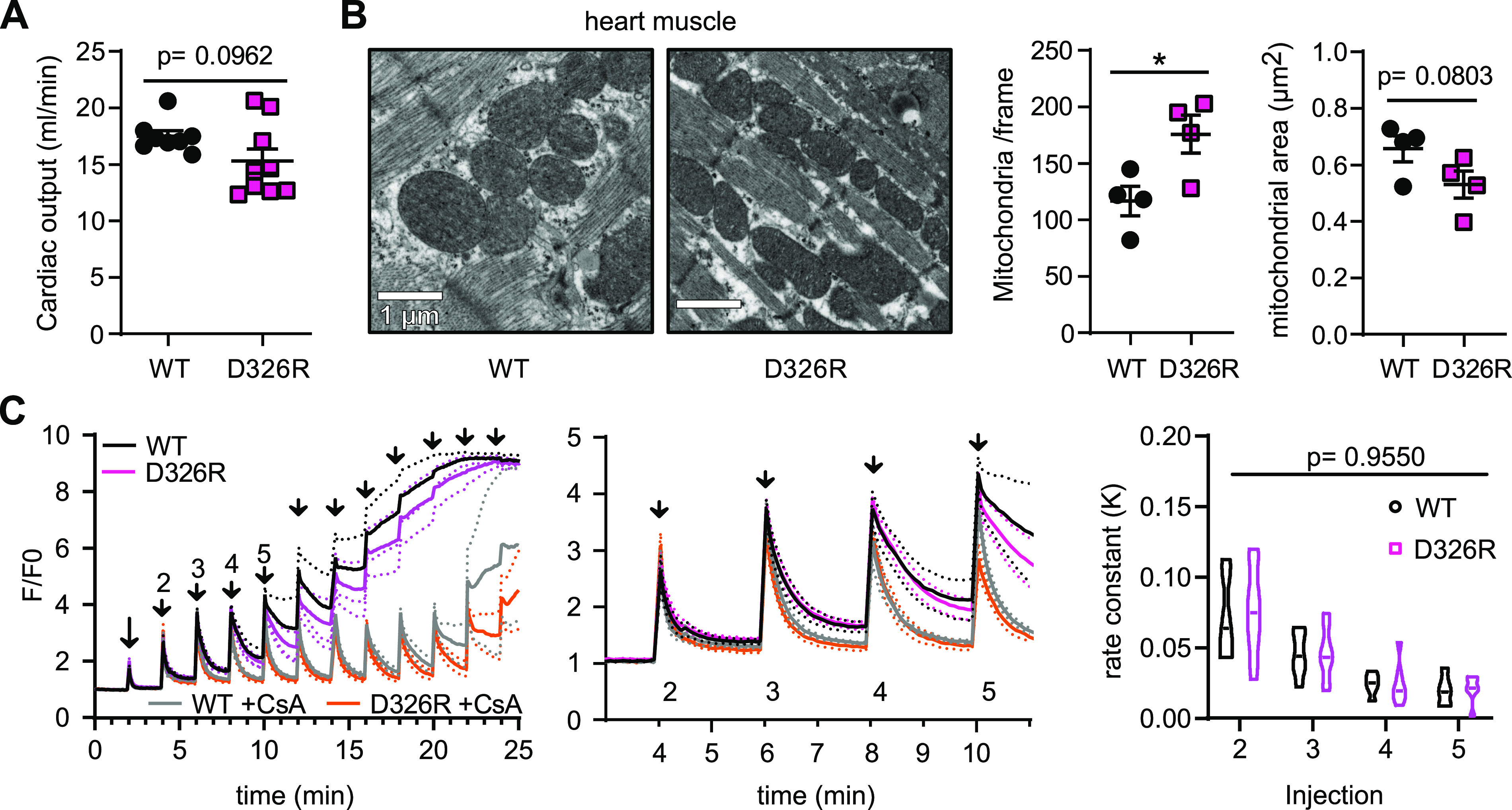
Normal heart function and increased number of normal appearing mitochondria in heart tissue of D326R mice. **(A)** Transthoracic echocardiography measured in parasternal long axis (PLAX) with analysis of the cardiac output. **(B)** Exemplary transmission electron microscopy images showing normally structured heart mitochondria with a decrease in size but an increase in abundance. **(C)** Normal Ca^2+^ uptake and uptake capacity in isolated TMBIM5 D326R in heart mitochondria. Bath Ca^2+^ was measured by Calcium Green-5N. When challenged with a series of Ca^2+^ pulses (10 μM each), mPTP in D326R heart mitochondria opens as in wild type. On the right, rate constant (K) of the uptake slope after the indicated Ca^2+^ injections ± CsA. Fluorescence was normalized to the initial value (F_0_). (WT ± CsA: 4/5; D326R ± CsA: n = 6). Data are shown as scatter blots with mean ± SE or violin blots indicating the median ± quartiles. **(A, B)** unpaired *t* test, (C) mixed-effects analysis, n.s. *P* ≥ 0.05, *P < 0.05.

In contrast to skeletal muscle, cardiac function assessed by measuring the ejection fraction using ultrasound was not significantly different between WT and TMBIM5 D326R mice (Fig 6A). Also, cardiac mitochondria did not display the disrupted cristae phenotype but were more abundant in mutant mice and tended to be smaller (Fig 6B). Ca^2+^ uptake and mPTP opening were also similar between WT and D326R mitochondria isolated from hearts (Fig 6C). We conclude that TMBIM5 and its ion transport function is tissue-specific and important for proper development and function of skeletal muscle but not cardiac muscle. The cristae phenotype in skeletal muscle mitochondria from TMBIM5 D326R mice coincided with early mPTP opening and a reduced Ca^2+^ uptake capacity in mitochondria obtained from these mice suggesting that the two observations are correlated.

### Increased basal swelling and susceptibility to K^+^-induced swelling in TMBIM5 D326R liver mitochondria

**Figure 7.**
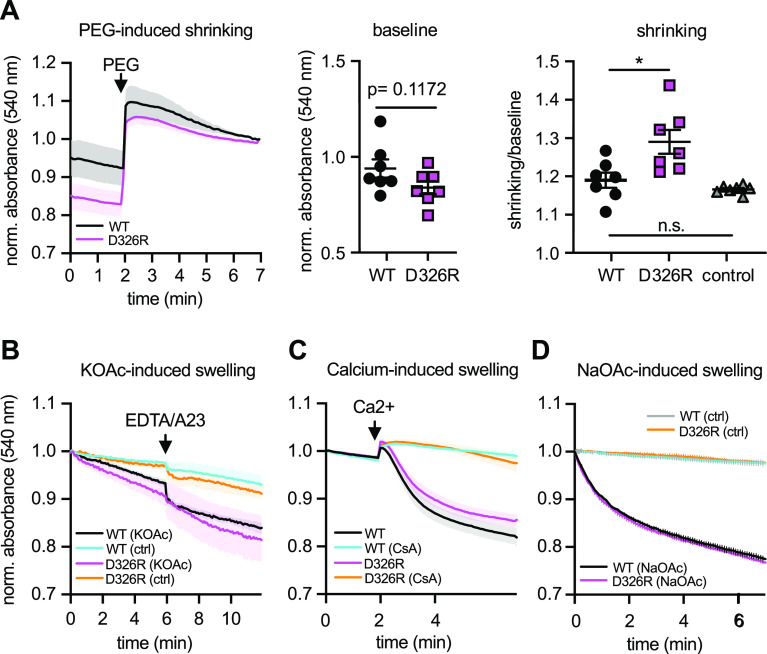
Increased swelling in TMBIM5 D326R liver mitochondria. **(A)** TMBIM5 D326R liver mitochondria showed a larger delta in absorbance after osmotic shrinking with polyethylene glycol (PEG, added where indicated). Values were normalized to the last values after PEG-addition. Baseline absorbance and the ratio of absorbance before and after shrinking are quantified on the right. **(B)** Spontaneous K^+^-dependent swelling of TMBIM5 D326R mitochondria. Mitochondria were firstly de-energized with 5 μM antimycin A. Then 10 mM EDTA and 1 μM A23187 (A23) were added to deplete mitochondria from divalent cations. A540 was followed immediately after the mitochondria were resuspended in the potassium acetate-based buffer (KOAc). Resuspension in the buffer served as control (ctrl). **(C)** TMBIM5 D326R mitochondria exhibit slightly reduced Ca^2+^-induced swelling. 200 μM CaCl_2_ was injected at the indicated time point to induce Ca2+ overload and mPTP opening. Inhibition of mPTP with 2 μM CsA served as control. **(C, D)** Na^+^-induced swelling did not differ between WT and TMBIM5 D326R. The mitochondria were de-energized as in (C) and resuspended in sodium acetate-based buffer and the swelling was immediately recorded. Acidification of the matrix led to Na^+^ influx and swelling. Resuspension in the buffer served as control (ctrl). Absorbance was measured at 540 nm and normalized to the initial value. All data are shown as mean ± SE, each data point corresponds to the mean of one individual experiment. **(A)** n = 7, unpaired *t* test, *P* is indicated, *P < 0.05. **(B)** n = 4, (C, D) n = 4–6. **(B, C, D)** Values were normalized to initial absorbance.

Based on the mitochondrial morphology observed by electron microscopy and the increased matrix K^+^ levels in TMBIM5 KO cells, we hypothesized that mitochondria of TMBIM5 D326R mice are already swollen at steady state because of an osmotic disbalance and that this renders them more susceptible to mPTP opening. We tested this assumption in mitochondria isolated from the livers of WT and TMBIM5 D326R mice by adding polyethylene glycol (PEG) to the mitochondrial suspension, which osmotically shrinks the mitochondria to their minimum size (Luongo et al, 2017). To compare the starting baseline, we normalized the absorbance to the value after PEG addition. This experiment revealed that D326R mitochondria are indeed pre-swollen (Fig 7A). We also examined differences in KHE activity between WT and D326R mitochondria. Mitochondria were de-energized by incubation with the complex III blocker antimycin A and KOAc medium added. Here, we observed that both WT and D326R mitochondria started swelling after the addition of KOAc. Whereas addition of EDTA and A23187 (Bernardi, 1999) resulted in the expected instant increase in swelling of WT mitochondria, mitochondria obtained from TMBIM5 D326R mice continued swelling as before (Fig 7B). In contrast to K^+^-induced swelling, Ca^2+^-induced swelling was rather reduced in D326R mitochondria (Fig 7C) in line with the reduced uptake capacity under certain conditions. Finally, swelling induced with sodium acetate (NaOAc) was indistinguishable between WT and D326R mitochondria (Fig 7D). We conclude that TMBIM5 D326R liver mitochondria are swollen at steady state and have an increased susceptibility to changes of K^+^ homeostasis. These results further support a function of TMBIM5 in the ion homeostasis of the mitochondrial matrix.

## Discussion

In this work, we studied the role of TMBIM5 in mitochondrial ion homeostasis. We found that overexpression of the mitochondrial protein TMBIM5 results in increased uptake of Ca^2+^ into the mitochondrial matrix upon agonist-induced IP_3_ receptor–mediated Ca^2+^ signalling. Under steady-state conditions, loss of TMBIM5 correlates with increased K^+^ and reduced H^+^ levels in the mitochondrial matrix caused by an attenuated exchanger activity resulting in osmotic disbalance. Mice that carry a point mutation in the channel pore display increased embryonic or perinatal lethality and a skeletal myopathy which strongly correlates with tissue-specific disruption of cristae architecture, early opening of the mitochondrial permeability transition pore, reduced Ca^2+^ uptake capability, and mitochondrial swelling.

Our results reveal TMBIM5 as a novel and yet unappreciated component of the mitochondrial ion transport system machinery affecting Ca^2+^ and K^+^ ions. A loose ion specificity has been described for other channel proteins where distinct ions are transported through the same channel protein depending on the driving force or specific conditions such as agonist stimulation versus basal conditions. Lysosomal TPC2 channels can switch their ion selectivity depending on the agonist (Gerndt et al, 2020) with being Ca^2+^ selective when opened by NAADP but Na^+^ selective when opened by PI(3,5)P_2_ (Patel et al, 2022). It is thus well possible that TMBIM5 can transport different ions depending on cellular needs and conditions. Homologues of TMBIM5 were described to permeate Ca^2+^ across ER membranes (Bultynck et al, 2012) and bacterial membranes (Chang et al, 2014), or Ca^2+^ and Na^+^ across lysosomal membranes (Pihán et al, 2021). K^+^ and Na^+^ ions at physiological concentrations can inhibit the Ca^2+^ binding ability of the prokaryotic TMBIM orthologue BsYetJ in vitro (Guo et al, 2019), implying different cations can be transported by TMBIM channel proteins in general.

TMBIM5 function affects Ca^2+^ and K^+^ ion homeostasis similar to LETM1, a putative Ca^2+^/H^+^ and K^+^/H^+^ exchanger. Both proteins are located in the IMM and have much in common. Their loss of function increases the matrix K^+^ concentration and results in ballooned mitochondria and a disrupted cristae architecture (Nowikovsky et al, 2004), both proteins are found in fungi, plants, and animals, and both are implicated in mitochondrial biogenesis. A recent proteome analysis of TMBIM5 KO HAP1 cells revealed a dramatic down-regulation of mitochondrial ribosomes and a reduction of proteins of the respiratory chain in these cells (Seitaj et al, 2020), similar to yeast lacking the LETM1 orthologue Mdm38 (Frazier et al, 2006). The ribosome binding domain of Mdm38 is distinct from its ion transport activity and this combination of ion transport and translational regulation apparently facilitates on-demand translation of mitochondrial membrane proteins by a direct coupling of the protein synthesis machinery with ion fluxes (Lupo et al, 2011). Also mammalian LETM1 associates with mitochondrial ribosomes (Durigon et al, 2018). Blue native gel analysis, however, revealed that TMBIM5 and LETM1 do not reside in the same macromolecular complex, although a very minor overlap could not be completely ruled out. TMBIM5 deficiency also did not affect the abundance of LETM1 suggesting that TMBIM5 works independently of LETM1. For LETM1, the central question of whether it works as a KHE or CHE has not been clarified since 2009, when Jiang et al (2009) proposed that LETM1 is a CHE (Jiang et al, 2009). Astonishingly, even the direction of Ca^2+^ transport is still unclear (reviewed in Natarajan et al [2021]). It therefore remains to be clarified whether TMBIM5 affects the correct functioning of the proposed KHE LETM1 or whether TMBIM5 is the ominous KHE itself. An ion channel function has been demonstrated in heterologous systems for LETM1 (Tsai et al, 2014; Shao et al, 2016) and for the bacterial TMBIM5 orthologue BsYetJ (Chang et al, 2014; Guo et al, 2019). Of all six TMBIM proteins, TMBIM5 and TMBIM6 are the closest homologues of BsYetJ (Lisak et al, 2015) making a direct channel activity of TMBIM5 very likely.

An open question is why the disruption of the channel pore and destabilization of the TMBIM5 protein by introducing the D326R mutation affected skeletal muscle so much more than the heart. Both tissues consist mainly of excitable cells that contract in response to changes in cytosolic Ca^2+^ concentrations. In both tissues, mitochondria are the predominant source of ATP and Ca^2+^ is the main link matching energy demand and ATP production in a feed-forward/feedback loop (Balaban, 2009; Schiaffino & Reggiani, 2011). Nevertheless, TMBIM5 loss of function resulted in changes of the cristae architecture, early opening of the mPTP and a reduced Ca^2+^ uptake capacity only in mitochondria obtained from skeletal muscle. Apparently, Ca^2+^ influx into skeletal muscle mitochondria exceeds the one measured in cardiac mitochondria by far presumably because the continuous cytosolic Ca^2+^ oscillations in cardiac cells put the mitochondria at a high risk of Ca^2+^ overload (Fieni et al, 2012). To prevent this, beat-to-beat Ca^2+^ influx into mitochondria has to be limited. For skeletal muscle mitochondria, in contrast, cytosolic [Ca^2+^] elevations are an important signal to enhance ATP generation. Hence, skeletal muscle mitochondria must be able to sequester considerable amounts of Ca^2+^ (Fieni et al, 2012). Paillard et al (2017) demonstrated that this is achieved by altering the stoichiometry of MCU and MICU1 in different tissues. The ratio affects both the threshold and uptake velocity of the MCU. A high ratio of MICU1:MCU ensures strict gatekeeping which results in a high Ca^2+^ threshold but also a higher maximal activity achieved by maximum MICU1 cooperativity (Paillard et al, 2017). In contrast, the abundance of MICU1 and the MICU1:MCU ratio is very low in cardiac tissue which correlates with a low threshold and a low maximal uptake rate (Paillard et al, 2017). Ca^2+^ uptake through the mtCU is additionally controlled by the integration of MCUb in cardiac mitochondria (Raffaello et al, 2013). MCUb is a dominant-negative paralog of MCU and its abundance relative to MCU is manyfold higher in the murine heart than in the skeletal muscle tissue (Raffaello et al, 2013). Moreover, in the skeletal muscle, a unique MICU1 splice variant (MICU1.1) was identified that increases the sensitivity of mtCU to Ca^2+^ and thereby induces uptake at lower [Ca^2+^] (Vecellio Reane et al, 2016). It is therefore well possible that either these players or similar changes specific to TMBIM5 (interaction partners, dominant-negative proteins or splice variants) govern the tissue specificity of TMBIM5 function.

In summary, we found that TMBIM5 affects mitochondrial ion homeostasis and the mitochondrial cristae architecture. This effect is most prominent in skeletal muscle mitochondria and its loss of function results in skeletal myopathy.

## Materials and Methods

### Colocalization analysis

Cells were seeded at the density of 100,000 in microscopy chambers (Ibidi). After 24 h, HEK293 cells were transfected as indicated (either with empty vector eGFP, TMBIM5-eGFP, TMBIM5^D294R/D325R^-eGFP, ^ΔMTS^TMBIM5-eGFP, or ^ΔMTS^TMBIM5^D294R/D325R^-eGFP). 48 h post-transfection, images were acquired sequentially at each laser excitations (405-Ar-561 nm) wavelength on a Zeiss LSM 880 – Airyscan. Z-stacks were recorded with a Plan-Apochromat 63x/1.4 Oil DIC M27 on living cells stained with MitoTracker Deep Red (Thermo Fisher Scientific, Cat. no. M22426) at the concentration of 100 nM in Krebs solution with 1.5 mM Ca^2+^ and glucose. HEK293 cells were incubated with 100 nM of MitoTracker Deep Red at 37°C for 30 min and washed twice with Krebs solution before confocal image acquisition. The microscope is equipped with temperature and CO_2_ controls, which were kept at 37°C and 10%, respectively. The cells were randomly chosen; two-color z-stack images were collected. A colocalization analysis was performed using JACoP (Just Another Colocalization Plugin) for Fiji. The colocalization was quantified using Manders coefficients calculated after manual determination of the threshold settings. The fraction of red (either MitoTracker Deep Red or mcherry-ER, kind gift from Prof. Patrizia Agostinis, KU Leuven) overlapping with the fraction of green (EV/TMBIM5/^ΔMTS^TMBIM5) was calculated for single cells and during three independent experiments. An average of 15 cells were analyzed per condition.

### Single cell Ca^2+^ measurements

Intracellular Ca^2+^ measurement in intact cells: HEK293 cells were loaded with 1 μM Cal-590-AM (AAT Bioquest) in the presence of pluronic acid (1:2,000) at RT in modified Krebs solution (150 mM NaCl, 5.9 mM KCl, 1.2 mM MgCl_2_, 11.6 mM Hepes [pH 7.3], 11.5 mM glucose, and 1.5 mM CaCl_2_) for 45 min. This was followed by a de-esterification step in the absence of extracellular Cal-590-AM for 30 min at RT. Extracellular Ca^2+^ was chelated with EGTA before stimulating cells with ATP (2 μM) or thapsigargin (TG, 2 μM; Sigma-Aldrich) to elicit intracellular Ca^2+^ release. Fluorescence (Ex 574/Em 588) was monitored using a Zeiss Axio Observer Z1 Inverted Microscope. Baseline was recorded for 30 s before the addition of Ca^2+^ triggers.

Mitochondrial Ca^2+^ measurements: Cells were co-transfected (either with empty vector eGFP, TMBIM5-eGFP, and ^ΔMTS^TMBIM5-eGFP) with the mitochondrial red Ca^2+^ indicator R-CEPIA3*mt* (Kanemaru et al, 2020), which has a Kd of 3.7 μM for Ca^2+^. pCMV R-CEPIA3mt was a gift from Masamitsu Iino (plasmid # 140464; Addgene). 48 h after transfection, cells were washed twice in modified Krebs solution (150 mM NaCl, 5.9 mM KCl, 1.2 mM MgCl_2_, 11.6 mM Hepes [pH 7.3], 11.5 mM glucose and 1.5 mM CaCl_2_). Fluorescence was measured with a Zeiss Axio Observer Z1 Inverted Microscope. Baseline was recorded for 30 s before addition of ATP.

### Denaturing immunoblotting

To obtain protein samples, cells were directly lysed in dodecyl-β-D-maltoside-lysis buffer (DDM-lysis buffer; 50 mM Hepes, 150 m NaCl, 0.2% DDM, 0.5 mM EGTA, and 0.3 mM CaCl_2_). Mouse tissue samples were homogenized for 30 s in the same buffer using a glass-Teflon-potter (IKA RW 16 basic) at 4,000 rpm (1-3x, until homogeneous). After 30-min solubilization (4°C, rotating), all samples were centrifuged (21,000*g*, 10 min, 4°C) and the supernatant was used for Western blotting. Protein samples were denatured in 1× Laemmli-β-mercaptoethanol-buffer, 95°C, 5 min. After gel electrophoretic separation of the proteins, they were transferred to nitrocellulose membranes by using a semi-dry blotting system (Bio-Rad). For the quantification of TMBIM5, membranes were incubated in SDS-β-mercaptoethanol solution (100 mM β-mercaptoethanol, 2% SDS, and 62.5 mM Tris–HCl, pH 6.7) at 55°C for 15 min on a shaker. After washing (TBST) and blocking (3% milk powder in TBST, 1 h, RT), the membranes were incubated with the respective primary antibodies (overnight, 4°C, rotating). Antibodies used: rabbit anti-TMBIM5 (Proteintech, 1:1,000), rabbit anti-MCU (Millipore Sigma, 1:500), mouse anti-OPA1 (BD Bioscience, 1:2,000), mouse anti-actin (Merck Chemicals, 1:1,000), rabbit anti-α/β-tubulin (Cell Signalling, 1:1,000), rabbit anti-coxIV (Cell Signalling, 1:1,000), rabbit anti-VDAC (Cell Signalling, 1:1,000). Fluorescence-labelled secondary antibodies were used, and the signal was detected using a Li-Cor Odyssey imaging system and quantified with the Image Studio Lite software. The intensity was normalized to the loading control and the mean per blot.

### Transmission electron microscopy

Tissue preparation and image acquisition were conducted by Ilse von Graevenitz (Institute for Microscopic Anatomy and Neurobiology, JGU Mainz). Mice were killed by cervical dislocation and the desired tissues were rapidly explanted and immersed in a fixative solution (2% paraformaldehyde, 1% glutaraldehyde in PBS). After fixation, tissues were washed in PBS with 3% saccharose followed by incubation in 0.1–2% OsO_4_ (in PBS) for 90 min. Tissues were washed again in PBS and then dehydrated in an ascending ethanol series and incubated overnight in 70% ethanol with 3% uranyl acetate. The next day, the samples were washed 3× with 100% ethanol and embedded in epon/glycid ether 100. Slices were cut using a Reichert Ultracut S ultramicrotome (Leica Microsystems) and contrasted with uranyl acetate and lead citrate. Images were acquired with a Zeiss - Leo 906e electron microscope at an acceleration voltage of 100 kV.

### Mitochondrial respiration

1 d before the experiment, 40,000 cells were seeded per well into 96-well cell culture microplates (Seahorse XF96, V3-PS, TC-treated; Agilent Technologies). The extracellular flux assay kit (Seahorse XFe96; Agilent Technologies) cartridge was hydrated with 200 μl XF Calibrant and sealed in a bag with a wet towel and left in a non-CO_2_ incubator overnight. The next day, cell confluency reached 90–100%. The cell plate was placed in the non-CO_2_ incubator at 37°C for 1 h before the experiment. The assay medium contained the Seahorse XF DMEM medium pH 7.4 (Agilent Technologies) supplemented with 2.5 mM glucose, 1 mM pyruvate, and 2 mM L-glutamine. Injection solution A contained a final concentration of 1 μm oligomycin, injection B 1 μM FCCP, injection C 1 μM rotenone, 1 μM antimycin A and 50 μg/ml Hoechst solution. The data were normalized to the cell number calculated by Hoechst staining at the end of the experiment.

### Mitochondrial Ca^2+^ flux and ER Ca^2+^ release in permeabilized cells

Mitochondrial Ca^2+^ flux was analyzed as described previously (Luongo et al, 2015, 2017). Briefly, WT and *TMBIM5*^*−/−*^ HEK293 cells were washed in Ca^2+^-free DPBS (Thermo Fisher Scientific, Cat. no. 14190235). An equal number of cells (7 × 10^6^ cells) were resuspended and permeabilized with digitonin (40 μg/ml) in 1.0 ml of intracellular medium (120 mM KCl, 10 mM NaCl, 1 mM KH_2_PO4, and 20 mM HEPES-Tris, pH 7.2), containing thapsigargin (2 μM) to block SERCA, and supplemented with 5 mM succinate. To monitor matrix-free Ca^2+^, MCU inhibitor Ru360 (1 μM) and NCLX inhibitor CGP37157 (10 μM) were also added. Fura-FF (1 μM) was loaded to the cell suspension, JC1 (800 nM) was added after baseline recording for 20 s, and fluorescence was monitored in a multiwavelength excitation dual-wavelength emission fluorimeter (Delta RAM, PTI). For ER Ca^2+^ release measurements, thapsigargin was added after basal Ca^2+^ recordings for 100 s. Extramitochondrial Ca^2+^ is shown as the excitation ratio (340 nm/380 nm) of Fura-FF fluorescence. Ca^2+^ boluses, Ru360, and mitochondrial uncoupler FCCP (2 μM) were added at the indicated time points. All the experiments were performed at 37°C with constant stirring.

### Steady-state mitochondrial Ca^2+^ levels

1 d before the experiment, 25,000 cells were seeded per well into black-walled 96-well microplates with clear bottoms (Greiner Bio-One, Cat. no. 655866) and transfected with pAAV-Syn2MT-mCherry-GCaMP6m (kind gift of Israel Sekler, Ben Gurion University [Stavsky et al, 2021]) using Lipofectamine. The next day, the medium was replaced with fresh medium containing 1 μg/ml Hoechst. Images were taken using the Opera Phenix high-content microscope with the 20× objective (1.0 NA) at 37°C and 5% CO_2_. The mCherry excitation was 561 nm and GCaMP6m at 488 nm. The images were analyzed using the Harmony software. To realize the automatic calculation of mitochondrial Ca^2+^ in the transfected cells, the following steps were conducted: (1) detected of individual cell/nucleus using the Hoechst channel; (2) based on this, then detected cytoplasm by mCherry channel; (3) calculated the cell size; (4) calculated the mean intensity of mCherry in the cytoplasm per cell; (5) selected the successfully transfected cells with the size in-between 90 and 400 μm^2^, and the mean intensity of mCherry over 300 au (based on the maximal value in the background which was measured manually by the “measure” tool); (6) calculated the mean intensity of GCaMP6m in the cytoplasm per cell; (7) calculated the ratio mean intensity GCaMP6m/mCherry; (8) gave the mean ratio of all selected/transfected cells per well. The HEK293T *MCU*-KO cell line was a gift from Kevin Foskett.

### Mitochondrial isolation for HEK293 cells

Three confluent 10 cm dishes of HEK293 cells were prepared and washed with cold PBS three times. Then 1 ml cold PBS was added to the dish and cells were scraped. Samples were centrifuged at 700*g* for 5 min at 4°C and the pellet was kept and resuspended in the isolation buffer (200 mM Sucrose, 10 mM Tris-MOPS, and 1 mM Tris-EGTA) and then transferred to a glass vessel for homogenization. Cells were lysed up and down manually at low speed for 5 min on ice, and drawn into a 5-ml syringe by 18 gauge 1–inch needle and expelled with 27 gauge 1/2-inch needle for five times. The needle tip was always against the wall to break the cell membrane when expelling. The samples were centrifuged at 600*g* for 5 min at 4°C and the supernatant was kept to get rid of the nuclei and intact cells. Afterwards, the supernatant was centrifuged at 10,000*g* for 5 min at 4°C and the pellet was resuspended in 50 μl isolation buffer. 1 μl of the mitochondrial prep was for BCA protein concentration measurement. The whole procedure was strictly done on ice. 600 μg protein of the isolated mitochondria were used for one mitochondrial PEG shrinking, K^+^ swelling experiments, and 100 μg protein for one mitochondrial sodium measurement.

### Mitochondrial K^+^ and Na^+^ quantification

To monitor mitochondrial K^+^, the specific probe NK1 was applied (Ning & Tian, 2020). HEK293 cells in a 10 cm dish were incubated in 1 μM NK1 in culture medium at 37°C for 10 min, and then washed with PBS twice. 1 × 10^6^ cells were suspended in 200 μl assay buffer (120 mM KCl, 10 mM NaCl, 1 mM KH_2_PO_4_, 20 mM Hepes, and 5 mM succinate, pH 7.2, treated via BT Chelex 100 resin) in a well of a black 96-well-plate and NK1 signal measured by Spark Tecan plate reader. The assessment of mitochondrial Na^+^ was adapted from Hernansanz-Agustín et al (2020). The cell-permeant sodium-sensitive probe benzofuran isophthalate tetra-ammonium salt (SBFI, S1263; Thermo Fisher Scientific) was applied, excited at wavelengths (340/380 nm) while emission at 500 nm. 100 μg protein mitochondrial prep was added up to 200 μl assay buffer in a well of a black 96-well-plate, with 5 μM SBFI, with or without 10 μM CGP-37157, incubating at 37°C for 30 min before the measurement at a plate reader.

### Mitochondrial pH quantification

HEK293 cells were transfected with pC1-SypHer3s-dmito, a mitochondrial matrix ratiometric pH probe with enhanced brightness for mammalian cells expression (Ermakova et al, 2018) (Plasmid #108119; Addgene) by Lipofectamine 1 d before. Transfection efficiency was above 90% shown by confocal microscopy. 1 × 10^6^ cells were suspended in 200 μl intracellular buffer (120 mM KCl, 10 mM NaCl, 1 mM KH_2_PO_4_, 20 mM Hepes, and 5 mM succinate, pH 7.2, treated via BT Chelex 100 resin), and the signals were measured by a Spark Tecan plate reader at 37°C. For the measurement of steady mitochondrial pH, the cells were intact. For the experiments with KCl supply: 40 μg/ml digitonin was additionally added before the measurement to permeabilize the cells; 10 mM EDTA and 1 μM A23187 were added to accelerate the KHE; 1 mM quinine was added to block KHE. Excitation 405 ± 20 nm (gain 80) or 488 ± 20 nm (gain 80), emission 535 ± 20 nm.

### Blue native polyacrylamide gel electrophoresis (BN PAGE)

Protein samples were solubilized with 5% digitonin on ice for 15 min followed by centrifugation (20,000*g*, 30 min, 4°C). 0.25% of G-250 was added to the supernatant and complexes were separated via 4–16% Bis-Tris gels (NativePAGE, Thermo Fisher Scientific). The complexes were transferred to PVDF membranes via wet blotting without methanol. After fixation (8% acetic acid), destaining (50% methanol and 25% acetic acid), and blocking (3% milk powder in TBST, 1 h, RT), the membranes were incubated with the respective primary antibodies (overnight, 4°C, rotating). The used antibodies are listed above. The staining of HRP-coupled secondary antibodies was detected with the Clarity Western ECL Kit (Bio-Rad)/SuperSignal West Femto Maximum sensitivity Kit (Thermo Fisher Scientific) using a Bio-Rad detection system.

### Mitochondrial swelling/shrinking assay

Mitochondrial swelling was detected as a decrease in the absorbance of isolated mitochondria in suspension. For Ca^2+^-induced swelling, Mitochondria were resuspended in assay buffer (125 mM KCl, 10 mM Hepes, 2 mM MgCl_2_, and 2 mM K_2_HPO_4_, pH 7.2 [KOH], freshly supplemented with 100 mM succinate and 0.2 μM thapsigargin) and after 2 min baseline a high concentration (liver: 200 μM, heart: 1 mM) of CaCl_2_ was added manually, which induced mPTP opening and mitochondrial swelling. Inhibition of swelling with Cyclosporine A (2 μM) served as control. For the Na^+^-induced swelling, mitochondria were suspended in the isolation buffer (200 mM Sucrose, 10 mM Tris-MOPS, and 1 mM EGTA-Tris, pH 7.4) and de-energized by incubation with 5 μM Antimycin A (10 min, RT). The buffer was removed by centrifugation (8,000*g*, 2 min), rapidly replaced by sodium acetate swelling buffer (55 mM NaOAc, 5 mM TES, 0.1 mM EGTA, and 0.1 mM EDTA) and the suspension was immediately transferred to a clear 96-well plate. Resuspension in the cellular buffer served as a negative control. The addition of the buffer with NaOAc directly started the swelling process. For both assays, the values were normalized to the initial values. As a control for pre-swelling, mitochondria were suspended in Ca^2+^-assay buffer and after a 2 min baseline, polyethylene glycol-3350 (PEG, final 5%) was added which induced shrinking of pre-swollen mitochondria. PEG is a highly viscous substance making it necessary to add a large volume (100 μl). As a control for the volume-induced change of absorbance, the same volume of assay buffer was added. All values were normalized to the last values after PEG addition which uncovered variations in the baseline state. For the K^+^-induced swelling, mitochondria were resuspended in a small volume of cellular buffer (120 mM KCl, 70 mM mannitol, 25 mM saccharose, 20 mM Hepes, and 5 mM KH_2_PO_4_, pH 7.5) and de-energized with 5 μM Antimycin A (10 min, RT). 9× volumes of potassium acetate swelling buffer (120 mM KOAc, 5 mM TES, 0.1 mM EGTA, and 0.1 mM EDTA, pH 7.4) were added and absorbance was measured. Addition of cellular buffer served as control. After 6 min, 10 mM EDTA and 1 μM A23187 were injected to deplete and chelate divalent cations. For the valinomycin-induced swelling, isolated mitochondria were resuspended in cellular buffer (120 mM KCl, 70 mM mannitol, 25 mM saccharose, 20 mM Hepes, and 5 mM KH_2_PO_4_, pH 7.5). Successively, 100 nM valinomycin or DMSO as vehicle and 5 mM succinate were added to induce energy-dependent swelling. For all assays, absorbance was measured at 540 nm using a plate reader.

### Generation of D326R mice

For generation of the C57BL6J-Ghitm^em1(D326R)LTK^ line, here referred to as D326R, we targeted exon 9 of the Ghitm gene. A 3 bp (GAT to CGC) mutation was introduced to obtain the amino acid change from D to R. In detail, starting from beginning of exon 9, we changed 5′-GATGTTGACAATCTACATGGATACATTAAA-3′ to 5′-GATGTTGACAATCTACATGcgcACATTAAA-3′. We used a guide with the sequence 5′-TAGGATGTTGACAATCTACA-3′ in combination with a single-stranded DNA (ssDNA) donor template with the sequence 5′-CACAAGCGGTTACTTCATTTCTTTCTGTTGCTTCCAGTTGCTAGCATAGTTGCAACTCGCATAAATATATTTAATGTgcgCATGTAGATTGTCAACATCCTAGAAAAAATATTACAGAT-3A′. Lyophilized CRISPR RNA (crRNA) and trans-activating CRISPR RNA (tracrRNA) (Alt-R CRISPR, iDT) were resuspended in 1× microinjection buffer (10 mM tris–HCl [pH 7.5] and 0.1 mM EDTA) to a final concentration of 10 μM. A total of 1.84 μl of tracr and crRNA was mixed with 10× injection buffer (5 μl) and 0.5 μl of *Streptococcus pyogenes* CAS9 protein (EnGen Cas9 NLS; 20 μM; New England Biosciences) and subsequently incubated for 15 min at 37°C. After incubation, the mix was diluted with double-distilled H_2_O to a final volume of 50 μl. For knock-in mouse production, 500 ng of single stranded DNA homology-directed repair donor (MWG) was added. The final mix was spun down at 21,000*g* for 3 min at RT. Injection mix was kept at RT during the injection procedure. Microinjection was performed at the transgenesis core of the University of Zürich, Institute of Laboratory Animal Science under license of the cantonal veterinary office (No. 177) in accordance with Swiss federal law. C57BL/6J mice at 3–4 wk of age (Charles River Laboratories) were superovulated by intraperitoneal injection of 5 IU of pregnant mare serum gonadotropin (Folligon, MSD Animal Health GmbH) followed 48 h later by injection of 5 IU of human chorionic gonadotropin (Pregnyl, MSD Animal Health GmbH). Mouse zygotes were obtained by mating C57BL/6 stud males with superovulated C57BL/6 females. Zygote microinjections, embryo culture, and retransfer into pseudopregnant foster animals were performed according to standard mouse transgenesis protocols (Harms et al, 2014). Founders were screened via PCR using the following primers: forward, 5′-GTTGCAACTATGCTAGCAAC-3′ and reverse, 5′-TAGGATGTTGACAATCTACA-3′, followed by Sanger sequencing. Sequence analysis was performed using the CLC Main Workbench software (QIAGEN). D326R mice were backcrossed to C57BL/6 and correct targeting was confirmed via sequencing before use in experiments.

### Animal experimentation

All animal studies were conducted in accordance with national guidelines and approved by the appropriate animal protection committees (23 177-07/G 18-1-026; Landesuntersuchungsamt RLP). For the inverted grid assay, mice were placed in the middle of a large grid which was then inverted and placed on top of a transparent cylinder (height: 25 cm, diameter: 17 cm). The latency to fall was measured. The observed was blinded to the genotype of the mice. For the echocardiography, anesthesia was induced in a chamber containing 2–4% isoflurane mixed with 0.2 liters/min 100% O_2_ and continued throughout the measurement via a face mask (1–2% isoflurane with 0.2 liters/min 100% O_2_). Animals were kept on a heated table mounted on a rail system (VisualSonics). Ultrasound was performed with the Vevo 3100 Imaging System (FUJIFILM Sonosite Europe) and the MX400 transducer (range: 20–46 MHz, center transmit: 30 MHz). The body temperature was monitored using a rectal probe and maintained at 37°C. The analysis was conducted using the software Vevo LAB 5.6.1 (FUJIFILM Sonosite).

### Isolation of mouse tissue mitochondria

To obtain functional mitochondria from mouse tissue, three different protocols were applied according to the tissue type (heart, brain, skeletal muscle). The mice were sacrificed by cervical dislocation, the desired organ was dissected and immersed in isolation solution (IS; 225 mM mannitol, 75 mM sucrose, 2 mM Hepes, and 1 mM EGTA, pH 7.4) (brain, heart) or basic medium (BM; 140 mM KCl, 20 mM Hepes, 5 mM MgCl_2_, and 5 μM EGTA, pH 7.0) (skeletal muscle). The rinsed tissue was then transferred to precooled tubes containing 500 μl (heart) or 1,000 μl (brain) BSA-proteinase-solution (1:10 Proteinase [1.6 mg/ml]: BSA [4 mg/ml]) or 500 μl homogenization medium (skeletal muscle) (HM; 140 mM KCl, 20 mM Hepes, 5 mM MgCl_2_, 1 mM EGTA, 1 mM ATP, 1% BSA, and 0.1 mg/ml Subtilisin A). The tissue was minced with scissors and manually homogenized on ice in precooled glass-Teflon potters for 7 min (heart, skeletal muscle) or with 2× 10× strokes (brain). 500 μl of BSA-proteinase-solution or 1 ml of HM wasadded to the heart and muscle homogenate, followed by another homogenization for 7 min. After centrifugation (heart: 500*g*, 5 min; muscle: 800*g*, 10 min; brain: 2,000*g*, 3 min; all centrifugation steps at 4°C), the supernatant was transferred to a new tube and centrifuged again (heart: 7,700*g*, 10 min; muscle: 12,000*g*, 10 min). After the high-speed centrifugation, the pellets of heart and muscle contained the mitochondria and were washed with 1.4 ml mitochondria suspension solution (MSS; 225 mM mannitol, 75 mM sucrose, 2 mM Hepes, and 20 μM EGTA, pH 7.4) (heart, 2×) or with BM (muscle, 1×). The final mitochondrial pellet was then resuspended in 100 μl MSS (heart) or 200 μl BM (muscle). For the brain, the pellet of the first low-speed centrifugation was resuspended in 1 ml BSA-proteinase-solution and centrifuged again at 2,000*g*, 3 min. The supernatant was centrifuged at 12,000*g* for 8 min. The mitochondria-containing pellet was resuspended in 100 μl MSS.

### Mitochondrial Ca^2+^ uptake assay in mouse tissue mitochondria

Mitochondrial Ca^2+^ handling was measured in the absence or presence of 2 μM cyclosporine A in freshly isolated mitochondria from WT and D326R mice. Isolation procedures as described above. PTP-EGTA-buffer (120 mM KCl, 70 mM mannitol, 25 mM sucrose, 20 mM Hepes, 5 mM KH_2_PO_4_, and 20 μM EGTA, pH 7.5) was used as experimental buffer. K-glutamate (5 mM) and Na-Malate (2.5 mM) were added freshly. The bath [Ca^2+^] was detected using the Ca^2+^-sensitive dye Calcium-Green-5N (1 μM). The signal was measured using a plate reader (Tecan M200 Pro) every 5 s, at Ex/Em 503/535 nm, at 37°C for 312 cycles (>400 cycles for brain). 10 μM CaCl_2_ diluted in PTP-buffer without EGTA was injected automatically every 2 min. For the analysis, the first read-out was defined as F_0_ and used for normalization. All traces are shown as F/F_0_.

## Supplementary Material

Reviewer comments
